# How does selfing affect the dynamics of selfish transposable elements?

**DOI:** 10.1186/1759-8753-3-5

**Published:** 2012-03-07

**Authors:** Thibaud S Boutin, Arnaud Le Rouzic, Pierre Capy

**Affiliations:** 1 LEGS, CNRS UPR9034, IDEEV FR3284, Avenue de la terrasse, Bat 13, 91198 Gif-sur-Yvette, France; 2 Université Paris-Sud, Orsay, France

## Abstract

**Background:**

Many theoretical models predicting the dynamics of transposable elements (TEs) in genomes, populations, and species have already been proposed. However, most of them only focus on populations of sexual diploid individuals, and TE dynamics in populations partly composed by autogamous individuals remains poorly investigated. To estimate the impact of selfing on TE dynamics, the short- and long-term evolution of TEs was simulated in outcrossing populations with various proportions of selfing individuals.

**Results:**

Selfing has a deep impact on TE dynamics: the higher the selfing rate, the lower the probability of invasion. Already known non-equilibrium dynamics (complete loss, domestication, cyclical invasion of TEs) can all be described whatever the mating system. However, their pattern and their respective frequencies greatly depend on the selfing rate. For instance, in cyclical dynamics resulting from interactions between autonomous and non-autonomous copies, cycles are faster when the selfing rate increases. Interestingly, an abrupt change in the mating system from sexuality to complete asexuality leads to the loss of all the elements over a few hundred generations. In general, for intermediate selfing rates, the transposition activity remains maintained.

**Conclusions:**

Our theoretical results evidence that a clear and systematic contrast in TE content according to the mating system is expected, with a smooth transition for intermediate selfing rates. Several parameters impact the TE copy number, and all dynamics described in allogamous populations can be also observed in partly autogamous species. This study thus provides new insights to understand the complex signal from empirical comparison of closely related species with different mating systems.

## Background

Transposable elements (TEs) are now considered as major components of genomes. These DNA sequences are able to invade, to multiply, and to spread across species despite their deleterious impact (on average) on their host. The capacity to generate structural and functional variability illustrates their impact on genome evolution [1-3]. If their ubiquity and their features can be only explained by selfishness [4,5], they are also able to enhance genome evolvability and to promote molecular exaptation. Their influence on genome evolution is supported by an increasing number of observations [6,7], including gene regulation *via *epigenetics landmarks [8-10], or the emergence of the V(D)J immune system in mammals [11].

The complex co-evolutionary dynamics between TEs and genomes was also enlightened by several population genetic models (see [12] for a review). Usually, the models assume that the number of copies grows to an equilibrium after a burst of amplification, this equilibrium being achieved when replicative transposition balances natural selection against insertions [13,14]. However, independent biological data on TE activity from several elements including *Alu *elements in humans [15], *copia *elements, and LTR retrotranposons in plants [16,17] or in *Drosophila *[18], as well as Class II elements [19], clearly show that the transposition rate is not constant. Indeed, transposition bursts are frequently observed. Changes in activity can be due to epigenetic factors, such as modified methylation patterns [20], and explain why some natural populations have variable TE activity. In any case, if an equilibrium could be reached on a short scale, maintaining such a status over long evolutionary stages seems unrealistic. Investigations based on simulations assuming random mating and accounting for parameters like copy activity, copy impact on host fitness, and mutation on TE activity [21,22], show that non-equilibrium dynamics are likely and predict cycles of re-amplification similar to those observed in 'Lotka - Volterra' prey-predator dynamics [23]. 'Molecular domestication' events (TE insertions reaching fixation in the host population because of their positive impact on their fitness) can also be reproduced *in silico *with models allowing a low rate of beneficial insertions [22]. By analogy, it was recently proposed that genomes could be seen as ecosystems in which TEs families are co-evolving species [24-26].

TEs being sexually transmitted parasites, almost all models assume random mating. Surprisingly, little is known on how TE copies behave in completely selfing species. Yet, hermaphroditism is widespread in the living world, particularly in plants, but also in about one-third of animal species [27]. Self-crossing enables individuals to reproduce on their own by self-fertilization; it generally induces inbreeding depression or decrease in genetic variation, but can also be advantageous when mates are scarce due to the rarefaction of the partners [28,29].

It is generally assumed that selfing has a strong functional impact on genomes and on their components, including selfish elements such as TEs [30]. During the last decade, a few models focusing on the impact of selfing on TE dynamics were developed [31,32]. As expected, they predict a decline of the probability of TE invasion due to reduced exchanges between selfers: the selfish invasion strategy of TEs relies on being transmitted to the offspring at higher frequency than expected under Mendelian segregation, and becomes ineffective in absence of outcrossing. So far, little is known about TE dynamics in partial selfers beyond the unrealistic assumption of the transposition-selection equilibrium [32].

Here, we propose to extend the existing theoretical corpus in two directions: (i) assessing the impact of intermediate selfing rates on non-equilibrium TE dynamics, and (ii) evaluating the influence of the mating system on the evolutionary properties of TEs and of their host species. To this end, the fate of TEs in populations with various selfing rates was followed *in silico *from the very first steps of the genome invasion to the long-term evolution (10,000 generations). In addition, we simulated the consequences of switching from an outcrossing to a self-fertilizing population.

## Results

TE dynamics from the first generation after introduction to their long-term evolution were explored by individual-based simulations. In a population of *N *diploid hermaphrodites, each parent can be a selfer with probability *Z*, and reproduces proportionally to its fitness *w *(see 'Methods' section, parameters of the model are summarized in Table 1). The selective impact *s*_*i *_of each TE insertion can vary from highly deleterious to advantageous according to the distribution probability of selective impacts (see [22]). A copy duplicates through replicative transposition (rate *u*), but can also be eliminated by deletion (e.g., excision with no reinsertion, or after ectopic recombination between two copies in direct orientation) with a rate *v*. Mutations occurring at rate *m *decrease the copy activity. Copies with no activity are non-autonomous, they are still *trans*-mobilizable; thanks to the transposition machinery produced by autonomous copies. In the simulations, only the elements able to transpose (i.e., autonomous and non-autonomous elements) were explicitly modeled, immobile relic copies are considered as deleted.

**Table 1:** Parameters of the model

Parameter	Definition
*N*	Number of individuals in the population
*Z*	Probability to reproduce by self-fertilization
*w*	Fitness of an individual
*n*	Number of TE copies in the genome
*s*_*i*_	Effect of insertion *i *on fitness
*μ*_*s*_	Average effect of TE insertions on fitness
*σ*_*s*_	Standard deviation of the effect on fitness
*P*_*s *> 0_	Proportion of TE insertions that improve the fitness
*u*	Replicative transposition rate
*v*	Deletion rate
*a*_*i*_	Activity of a copy *i *(ability to produce the transposition machinery)
*m*	Mutation rate (toward a less active copy)
*σ*_*m*_	Standard deviation of the effect of a mutation on activity

### Population invasion

**Figure 1:**
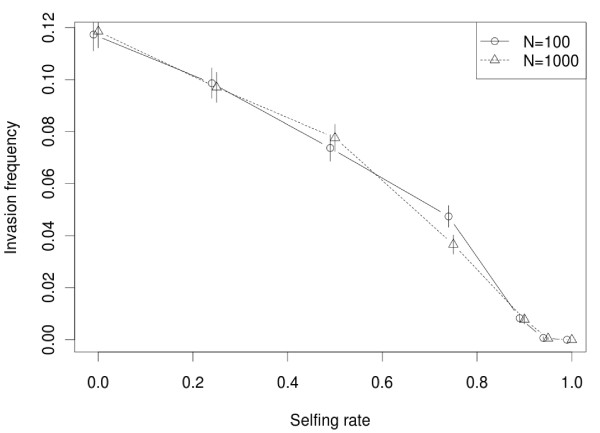
**Effect of selfing rate on the probability of invasion**. Initially, each population has only one full active copy. The probability of adaptive insertion was fixed to *P*_*s *> 0 _= 0.1%, this parameter does not affect the pattern (data not shown). The *Y *-axis is the percentage of invasion achieved (the frequency of the simulations with at least 1 copy per individual on average at generation 100), and the *X*-axis is the selfing rate (*Z*). Two population sizes were tested (*N *= 100 and *N *= 1000). The error bars represent the maximum-likelihood 95% confidence intervals, calculated assuming a binomial distribution of the invasion probability.

A population was considered as invaded by the TE family when one copy per individual on average is present 100 generations after the initial introduction. Copies spread from their single ancestor during an initial burst of transposition, when most of the copies are highly active. This invasion is expected to be facilitated by outcrossing [33]: the more selfing, the lower the probability of invasion. This is illustrated in Figure 1 for two population sizes. In addition, the distribution of copy number greatly varies according to the selfing rate. After 100 generations, when *Z *= 0.9, most individuals from successfully invaded populations have a single copy, while when *Z *= 0 (i.e., random mating), the average copy number per individual is much higher. Examples of the copy number distribution per individual after 100 generations are provided in Additional file 1.

On one hand, when the selfing rate is high (*Z *≥ 0.9), the invasion frequency is very small after 100 generations. However, in successfully invaded populations, active copies can be maintained for a while (up to 1,000 generations, data not shown) with apparent bursts in the 100 first generations (Additional file 2). These bursts do not correspond to an increase in copy number in the whole population, they are rather due to the accumulation of homozygous copies in some lineages until selection removes them. The invasion of a TE family is a stochastic process and is partly driven by genetic drift, but contrary to the well-known influence of population size on the fixation probability of deleterious mutations, the invasion frequency of TEs is virtually insensitive to the number of individuals. This effect, already documented in [34], is due to the deterministic aspect of TE invasion above a copy number threshold in the population, which does not depend directly on the population size. Moreover, there is no or little effect of the probability of adaptive insertion during the first steps of invasion whatever the selfing rate: such rare insertions are unlikely to happen soon enough to influence the early dynamics (data not shown).

### Beyond the initial burst

**Figure 2:**
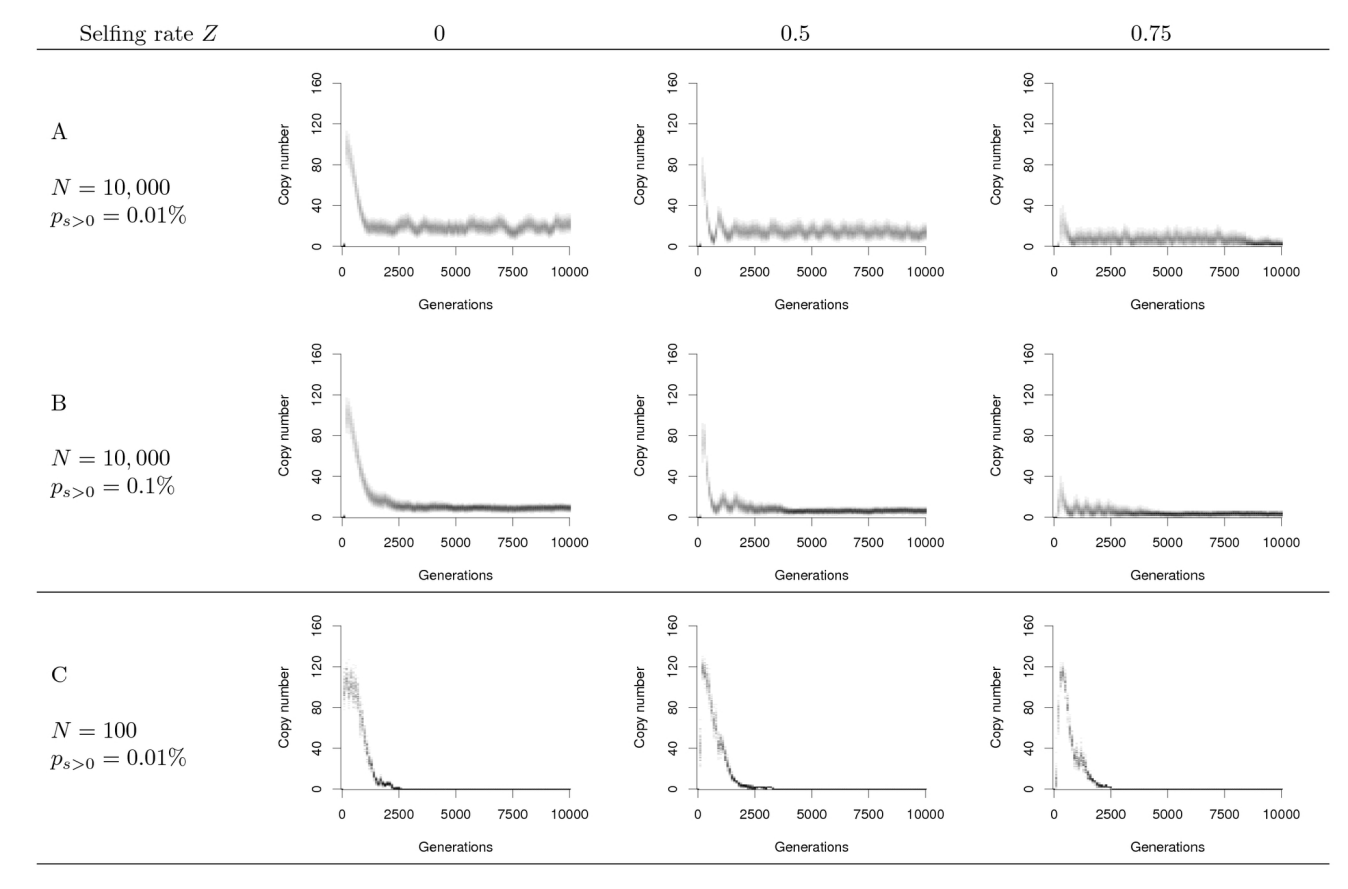
**Effect of selfing on the long-term dynamics after invasion of the initial copy**. Representative dynamics of the long term invasion (10,000 generations from 1 initial copy within large and small populations (*A, B *: *N *= 10,000 and *C *: *N *= 100) and different selfing rates *Z *= 0, *Z *= 0.5, *Z *= 0.75). A single simulation is presented for each case. The level of gray is proportional to the frequency at which each copy number is present in the population. The initial burst of transposition is followed by a decrease in the number of TEs due to the emergence of non-autonomous copies. Active transposition can persist when the probability of adaptive insertion is low *P*_*s *> 0 _= 0.01% **(A) **and 'domesticated' copies can be fixed **(B) **when this probability is high (*P*_*s *> 0 _= 0.1%). The frequency of cycles tends to increase with the selfing rate for any value of the probability of adaptive insertion. In opposition to large population size, the loss of the TE family is systematic in small populations **(C)**.

In the absence of a copy number control mechanism, the initial invasion may drive the host population to extinction, owing to the genetic load generated by a growing number of deleterious insertions [34]. In our model, the decrease in copy number is due to their elimination by natural selection and to a reduction of the general activity of transposition (regulation). After the initial burst, TE dynamics may go through cycles of re-invasion, molecular domestication, or loss (Figure 2), in the same way as for random mating populations [22]. Stable transposition-selection equilibria were not observed with the tested set of parameters.

The accumulation of mutations leads to the emergence of low-activity and non-autonomous copies. The reduction of the global transposition activity is due to the decline of the production of efficient transposition machinery. As long as the average activity in a genome is non-null, both autonomous and non-autonomous copies can transpose. In such a situation, the non-autonomous copies parasitize the autonomous elements, and cycles of decline/re-invasion can be maintained up to the end of the simulation (10,000 generations). Previous analysis has shown that these cycles can be explained by interactions between autonomous and non-autonomous copies, similarly to prey-predator (or host-parasite) interactions [22,25]. Autonomous copies are equivalent to hosts, as they are able to spread and maintain by themselves, but non-autonomous copies act as parasites: they can transpose only when autonomous copies are present in the same genome, forcing them to share the transposition machinery.

Figure 2A and 2B show that the frequency of the cycles increases with the selfing rate in large populations (*N *= 10,000). This phenomenon is clearer when cycles are maintained over a long period, e.g., when the probability of adaptive insertion is relatively low (Figure 2A), but it is still detectable when this probability is high (*P*_*s *> 0 _= 0.1%) before the fixation of adaptive insertions (Figure 2B). When the selfing rate increases, the fixation of adaptive insertion is delayed, while the copy number is about the same at the top of the first peak (comparison between *Z *= 0 or *Z *= 0.5).

As the probability of adaptive insertions increases, advantageous elements can be fixed more often (Figure 2B), provided that their positive effect is strong enough to overcome genetic drift. These beneficial insertions do not need to maintain their transposition activity to survive, and they eventually become inactive. The presence of a substantial amount on non-autonomous copies that are not purged by natural selection or deletions prevents the reinvasion of autonomous copies as observed in the cyclic dynamics, and transposition activity tends to be lost. At the end of this 'molecular domestication' process, other neutral and deleterious copies are progressively eliminated from the genome by selection, deletion, and drift, leading to the maintenance of few adaptive insertions that are totally inactive (*a *= 0).

Molecular domestication is consistently observed when the probability of adaptive insertion is high (*P*_*s *> 0 _= 0.5%, data not shown). For intermediate values, the proportion of domestication increases with the probability of adaptive insertions, and decreases with the selfing rate. Selfing populations also tend to fix less adaptive insertions on average.

The analysis of the joint effect of selfing and population size is also interesting. Indeed, while selfing has a clear effect on the long-term evolutionary dynamics of TEs in large populations (*N *= 10, 000), its impact is lowered by genetic drift in small populations (*N *= 100), in which TEs are generally lost after few thousand generations whatever the mating system (Figure 2C). Indeed, genetic drift also affects TE sequences, and increases the chances to lose all active copies in the population when the average copy number is low. This observation confirms previous findings [22], and highlights a mechanism acting in a direction opposite to the improved purging efficiency in large population-size species [35,36].

### Transition to selfing

**Figure 3:**
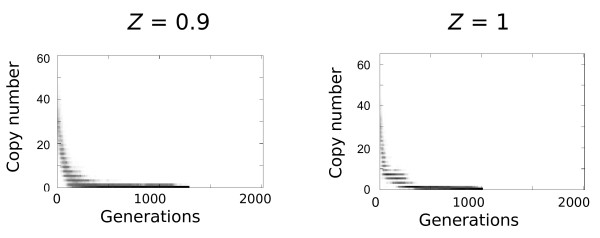
**Effect of switching from outcrossing to self-fertilization**. The two figures represent typical dynamics of a TE family simulated within a selfing population which has outcrosser ancestors. The initial population was originally a strict outcrossing population (*N *= 2000), already invaded by TEs, in which an abrupt transition to self-fertilization is performed. The new population thus has a historical background with numerous copies, both autonomous and non-autonomous, and few domesticated insertions (*P*_*s *> 0 _= 0.01%). Very rapidly after the transition to selfing (generation 0), almost all insertions become homozygous (even number of copies in all lineages). TEs are then systematically lost after few hundred generations, including adaptive insertions.

A family of TEs could invade a population with a very high selfing rate but has low chances to reach a high copy number if they are deleterious. However, an interesting situation can be found when a TE-rich outcrossing population switches to selfing. This was simulated with an abrupt transition in the mating system after the initial transposition burst. A few generations after the shift, the selfing population is *de facto *subdivided into competing lineages with slightly different TE composition (Figure 3). The lineages with the lowest copy number can persist longer. Finally, all TEs are lost after a few thousand generations. The loss remains a stochastic process, and in small populations (*N *= 100), the mean number of TEs can increase stochastically within lineages for several hundred generations, but active TEs always disappear eventually.

## Discussion

### Model interpretation

Our results confirm that the mating system has a deep impact on TE dynamics. Most of the main dynamics already described in random-mating populations were found. Depending on the properties of the TE family and of the host population (including population size and selfing rate), the initial burst can be followed by the loss of all copies, by the fixation of a few beneficial insertions (molecular domestication), or by cycles of reinvasions. As in random-mating populations, the population size has little influence on the first steps of TE invasion [34]. However, the TE dynamics in small populations are less stable, and lead more often to the loss of TE activity. Partial selfing affects the outcome of the model, and as a general rule, TEs are less likely to maintain (either as active parasites or as 'domesticated' insertions) in selfing populations. The probability of invasion decreases with the selfing rate, but when it occurs, the initial burst of transposition remains comparable to outcrossers (except for a slight delay), followed by one of the typical dynamics (cycles, loss, or domestication), depending on the mutation rate and the frequency of beneficial insertions. Thereby, the efficiency of TEs as genomic parasites decreases regularly with the selfing rate, but rare successful invasions can still occur even in populations with 90% selfers.

The model on which our simulations are based is original, and provides significant novelties compared to the existing literature: (i) the reproduction mode is flexible and allows partial selfing; (ii) TEs are affected by mutation, which generates inactive copies, similar to those found in sequenced genomes; (iii) transposition regulation occurs spontaneously as a consequence of copy polymorphism (the transposition rate depends on the ratio between autonomous and non-autonomous copies); and (iv) simulations are initiated from a single copy, instead of TEs artificially distributed among individuals. In previous study, dealing with diploid random mating populations (e.g., [22]), short- and long-term evolution was generally disconnected. Indeed, long-term simulations were initiated with many TE copies (often one copy per individual on average), so that the element cannot be immediately lost by drift. In this study, these two stages of the TEs dynamics are not disconnected and simulations were followed from the initial introduction of a single copy up to 10,000 generations. Our results being similar to those previously published for the same sets of parameters, disconnection of short- and long-term evolution seems to have little effect on the dynamics observed, which strengthens previously published work.

For modeling purposes, the system had to be simplified. For instance, only mobile copies are tracked, and the loss of mobility is assimilated to deletion. As a result, some populations may quickly lose all of their TEs in the simulations, while their genome may still contain relic copies. Our purpose being to investigate the influence of sexual reproduction, both transposition and deletion rates were maintained constant (*u *= 0.1, *v *= 0.001, respectively). These figures are quite high compared to empirically measured rates (e.g., [37]), but dynamical parameters are known to scale the dynamics of the process without affecting the pattern [22,38] (i.e., decreasing the transposition rate slows down the dynamics by the same order of magnitude). Interestingly, the reproduction mode itself might influence transposition parameters. For instance, it is likely that ectopic recombinations (and thus opportunities for TE deletion) are less frequent in selfers. Our model did not consider such complex mechanisms, but this evidences how simplistic our understanding of TE dynamics remains.

An interesting observation is that amplification-loss cycles, suggested by genomic data and described theoretically in [23], can be also observed in autogamy, but with a higher frequency (Figure 2). This accelerated evolution can be attributed to the non-random distribution of TE copies among individuals in partial selfers: as illustrated in Additional file 1, the variance in copy number (and thus the variance in fitness) increases with selfing (the distribution is wider than the expected Poisson under random mating), thus increasing the efficiency of natural selection and accelerating the elimination of TEs. The number of 'domesticated' copies is also lower in selfers: in absence of recombination (or in exclusively homozygous individuals after several generations of selfing), adaptive insertions always remain associated to deleterious elements, which prevents their spread, according to the Hill-Robertson effect [39,40].

Our results also highlight the consequences of a shift from outcrossing to selfing, a frequent evolutionary transition among hermaphrodite flowering plants [41]. Such an event increases homozygosity, and subsequently leads to the formation of divergent lineages with variable TE copy number. As a consequence, the variability in TE content (and accordingly, the variance in fitness) is much larger between lineages than within lineages, explaining the rapid loss of TEs in the simulations. Nevertheless, this model does not explain why some selfing species still maintain active TEs in their genomes.

### Confrontation with data

Models of TE dynamics predict that selfish genetic elements should be unable to invade the genome of autogamous or asexual host species. We have also shown that TE copies should rapidly be lost after a shift from outcrossing to selfing. However, this prediction is not strongly supported by empirical data. Indeed, TEs are a major component of plant genomes, including selfing species such as the common wheat *Triticum aestivum*, whose genome is made of about 80% of TEs [42-44]. In addition, the comparison of the distribution of some TE families in *Arabidopsis *genus remains inconclusive. As expected, the self-fertilizing *Arabidopsis thaliana *has less polymorphic TE insertion sites than its outcrossing sister-species *Arabidopsis lyrata*, and also displays less TE copies in total [45,46]. However, the contrast remains much weaker than predicted by theory.

Several non-exclusive mechanisms have been proposed to explain the discrepancies between theory and observations. Recurrent horizontal transfers of active elements in self-fertilizing or asexual species could partly explain how genomic parasites are maintained in spite of unfavorable mating systems. Ancient asexual species are rare, as they are expected to be outcompeted by more evolvable sexual relatives, but an interesting exception can be found in Bdelloid Rotifers, in which asexual lineages are maintained for tens of millions years partially because of massive horizontal genes transfers compensating the absence of sexual genetic exchanges [47]. TEs were part of this alien DNA material, and some of them have slightly been amplified since these animals display a low content in potentially active repeated TE sequences [48]. In fact, TEs are more likely to be laterally transferred than any other genetics elements because they are fully equipped for genomic mobility. Such a phenomenon seems to have an important role in the spread of TEs between Eucaryotes [49], and horizontally transmitted TEs have been found in both animals [50-53] and plants such as the genus *Oryza *that includes selfing species [54,55].

Another explanation for the weak contrast between selfing and outcrossing genomes lies in the nature of the deleterious effect of repeated sequences [56]. While heterozygous insertions are likely to be involved in potentially catastrophic ectopic recombinations, homozygous copies may be closer to neutrality. TEs could thus be protected from natural selection in mostly homozygote selfers [31,32,57]. If biologically important, this phenomenon should be detected by comparing the TE-content of autosomes versus X chromosomes (hemizygous in males), expected to display less copies. Unfortunately, X chromosomes also experience lower recombination rates, which has the opposite effect [58]. Here again, the evidence is thus equivocal: homozygosity actually reduces ectopic recombinations in *Drosophila melanogaster *[59], but the X chromosome of mice and humans is particularly rich in *L 1 *TE sequences [60,61]. In addition, other mechanisms such as X inactivation may interfere with the expected pattern. The recent comparison between the genomes of *A. lyrata *and *A. thaliana*, in which the proportion of TE is lower, does not suggest selection against ectopic recombinations, but rather supports a selection against negative impact of insertions on surrounding genes [62].

Whatever is the focus of the natural selection, its efficiency depends directly on the effective host population size (*N*_*e*_). In a full selfing population, *N*_*e *_is reduced by half [63]. It was recently argued that population process could be a key factor in the patterning of TE distribution in plants [64]. The rare empirical population studies suggest a demographic impact (more copies in bottle-necked populations) among plants like *Arabidopsis *[45,65], but also in animals like nematodes [66]. Finally, observed differences might be due to non-deterministic factors: non-equilibrium dynamics are common in our simulations, and two species considered at two different stages of their cyclical dynamics may differ considerably in their TE content. In general, genetic drift may also play an important role in the persistence of active copies, as shown here and previously [22].

## Conclusion

In this article, our intention was to determine, using a realistic individual-based model, how TEs evolve in species with different selfing rates. We confirmed the well-known result that selfing tends to reduce TE content, by preventing the invasion, and by decreasing the number of copies. The dynamics described in outcrossing species are also found in partly self-fertilizing ones, but the details of the invasion process may be significantly affected. Interestingly, TE invasion remains possible even at high selfing rates. Our models also suggest that automagous populations fail at fixing rare beneficial insertions. Because selfing lessens by several ways the potentially beneficial mutagenic impact of TE copies, our results strengthen the catastrophic evolutionary consequences of the loss of sexual reproduction. If the mating system changes from outcrossing to selfing, the genome content in TE is strongly affected. However, sequenced genomes show that species classified as self-fertilizing often maintain a significant amount of active TEs, which contradicts the theory. Among other explanations, this observation suggests that species classified as selfers might experience rare outcrossing events, hindering the eradication of their genomic parasites. Moreover, horizontal transfers can play an important role in the maintenance of TE-mediated evolvability in selfing lineages.

## Methos

### Transposable elements

The model used for the simulations is individual-based and stochastic, inspired from the framework described in [22]. Within a genome, each copy can be duplicated with a maximum rate of transposition *u *= 0.1, and may be excised with a rate *v *= 0.001. In addition, each copy is defined by its own activity *a*_*i*_, i.e., the capacity of the element *i *to produce a functional transposition machinery. Thus, the effective rate of transposition is u⋅ā, where  is the mean activity of all (*n*) TE copies of a genome defined as $ā=\frac{1}{n}\sum a_{i}$. Mutation of copy activity occurs with a rate *m *= 0.001. The activity of a mutated element is $a_{i}^{\prime }=a_{i}-\left|\delta \right|$, where $\delta \sim N\left(\mu =0,\sigma_{m}=1\right)$. On average, it thus takes one or two mutations to turn a fully active element (*a*_*i *_= 1) to an inactive copy (*a*_*i *_= 0), which is still *trans*-mobilizable. Here, we did not consider the possibility for an element to lose its mobility in presence of transposition machinery: mutational events leading to immobilization are associated with deletions (the element disappears from the pool of observable copies).

### Host population

The population is based on a constant number of *N *diploid hermaphrodites with a genome made up of 5 chromosomes of 100 cM each. Throughout the non-overlapping generations, individuals are able to reproduce proportionally to their fitness *w*. The fitness is defined by *w *= 1 + Σ *s*_*i*_, where $s_{i}\sim N\left(\mu_{s}=-0.01,\sigma_{s}\right)$ is the selective impact of the insertion *i*. The variance $\sigma_{s}^{2}$ of this distribution conditions the probability of adaptive insertions, $P_{s_{i}}>0$ (see [22] for more details). *Z *being the selfing rate, each offspring has a probability *Z *of having a unique parent (which provide two gametes) and a probability 1 *- Z *of having two parents randomly drawn in the population, each of them providing one gamete. Gametes are generated by drawing the number of recombinations in a Poisson distribution, spreading the recombinations out uniformly in the genome, and copying randomly one of the chromosomes until a recombination is met, at which point the copy continues from the homologous chromosome. The recombination rate is the same in selfers than in outcrossers.

### Simulations

In most of the simulations, a single fully active copy was introduced in one of the individuals of the population. The dynamics of the element was followed from generation 1 to 10,000. To analyze the transition from cross fertilization to strict self-fertilization, the set of parameters used was one of those which led to a cyclic dynamics (see legend of Figure 2). Then, the mating system was switched (from *Z *= 0 to *Z *= 0.9 or *Z *= 1) at the top of the first wave.

## Abbreviations

LTR: long terminal repeats; TE: transposable element.

## Competing interests

The authors declare that they have no competing interests.

## Authors' contributions

TSB and PC designed the research. TSB and ALR carried the research out and analyzed the data. ALR conceived the core software. TSB, ALR, and PC interpreted the data and wrote the article. All the authors read and approved the final manuscript.

## Supplementary Material

Additional file 1 **Distribution of the copy number at generation 100**. This figure shows the distribution of copy number per individual at the 100^th ^generation for different selfing rates in a small population (*N *= 100). Click here for file

Additional file 2 **First step of invasion**. This figure shows the dynamics of TEs invasion for different selfing rates in a small population (*N *= 100). A single simulation is presented for each case. The level of gray is proportional to the frequency at which each copy number is present in the population at each generation. Click here for file
