# Supplementary figures and images for: How does selfing affect the dynamics of selfish transposable elements?
Source: Mob DNA. 2012 Mar 7;3:5. doi: 10.1186/1759-8753-3-5 (PMC3395816; doi:10.1186/1759-8753-3-5)

# Supplemental material

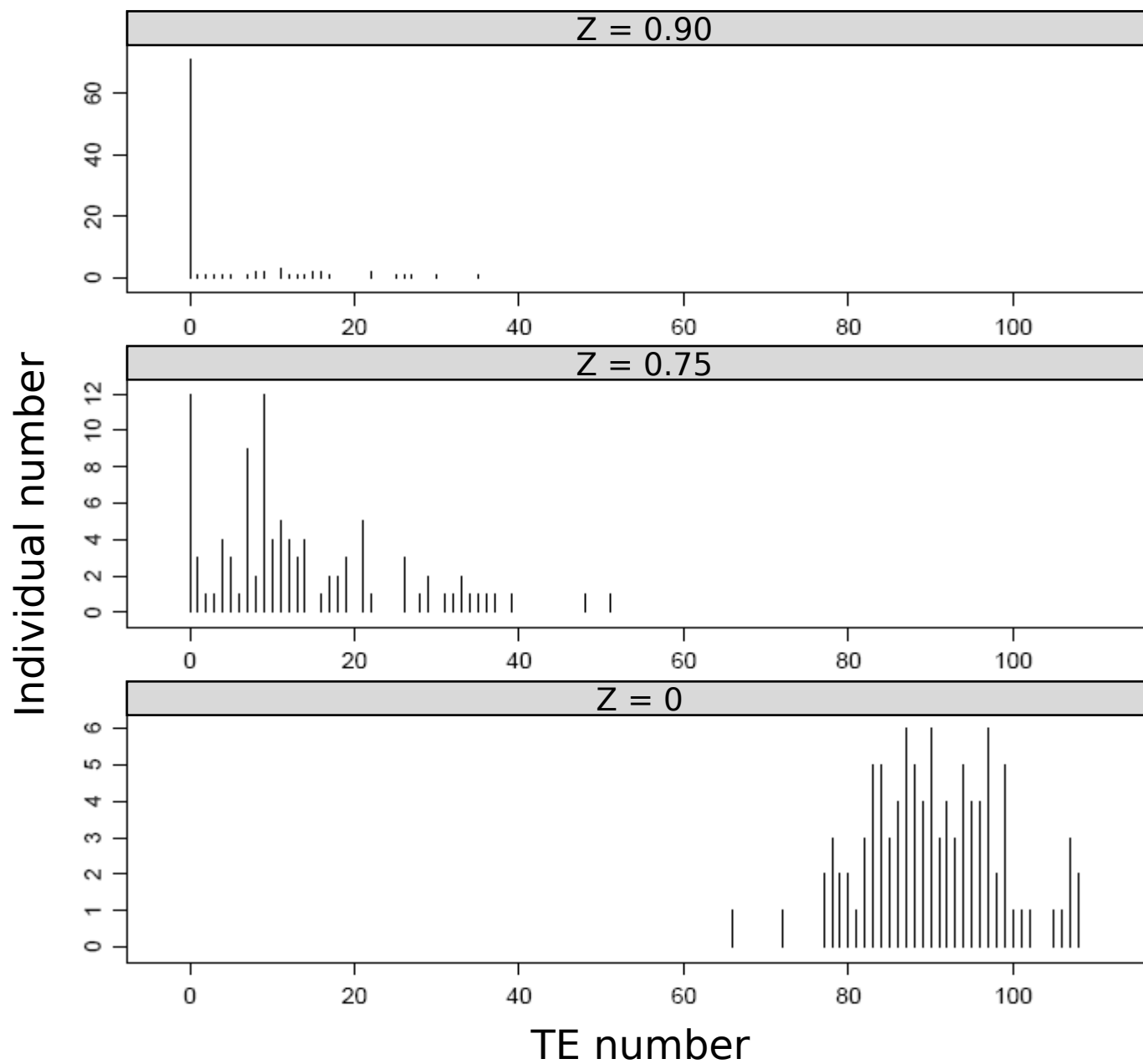

Figure S1

Supplement: Additional file 1 — Distribution of the copy number at generation 100. This figure shows the distribution of copy number per individual at the 100th generation for different selfing rates in a small population (N = 100). [file 1759-8753-3-5-S1.PDF]

# Supplemental material

$Z = 0$

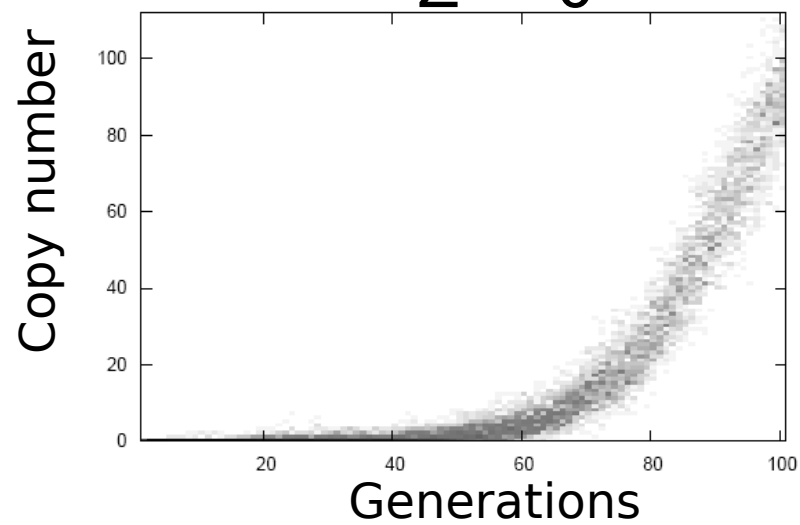

$Z = 0.75$

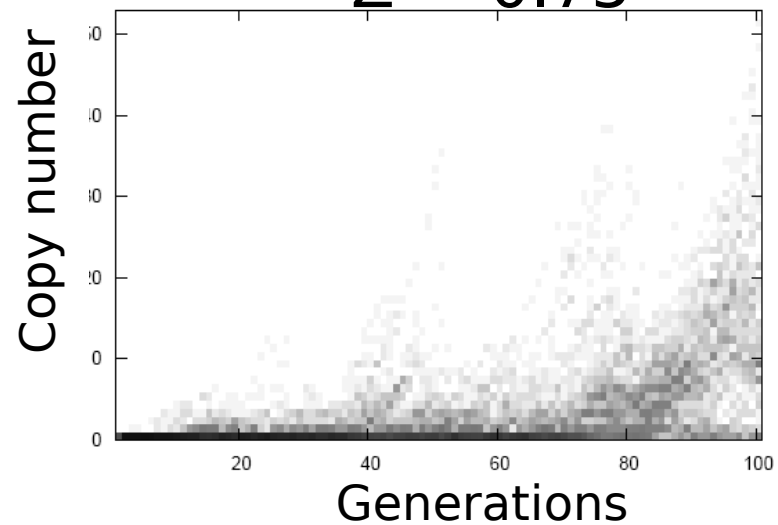

$Z = 0.9$

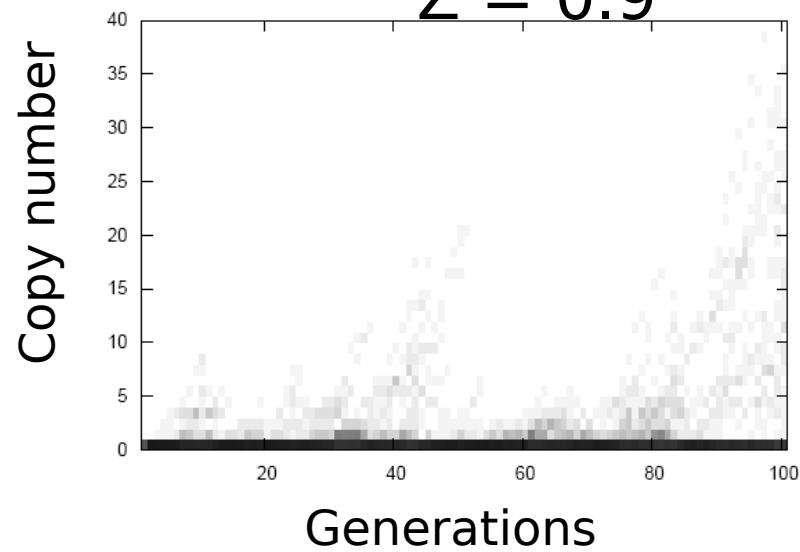

Figure S2

Supplement: Additional file 2 — First step of invasion. This figure shows the dynamics of TEs invasion for different selfing rates in a small population (N = 100). A single simulation is presented for each case. The level of gray is proportional to the frequency at which each copy number is present in the population at each generation. [file 1759-8753-3-5-S2.PDF]
